# Probability matching in perceptrons: Effects of conditional dependence and linear nonseparability

**DOI:** 10.1371/journal.pone.0172431

**Published:** 2017-02-17

**Authors:** Michael R. W. Dawson, Maya Gupta

**Affiliations:** Department of Psychology, University of Alberta, Edmonton, Alberta, Canada; University of Sheffield, UNITED KINGDOM

## Abstract

Probability matching occurs when the behavior of an agent matches the likelihood of occurrence of events in the agent’s environment. For instance, when artificial neural networks match probability, the activity in their output unit equals the past probability of reward in the presence of a stimulus. Our previous research demonstrated that simple artificial neural networks (perceptrons, which consist of a set of input units directly connected to a single output unit) learn to match probability when presented different cues in isolation. The current paper extends this research by showing that perceptrons can match probabilities when presented simultaneous cues, with each cue signaling different reward likelihoods. In our first simulation, we presented up to four different cues simultaneously; the likelihood of reward signaled by the presence of one cue was independent of the likelihood of reward signaled by other cues. Perceptrons learned to match reward probabilities by treating each cue as an independent source of information about the likelihood of reward. In a second simulation, we violated the independence between cues by making some reward probabilities depend upon cue interactions. We did so by basing reward probabilities on a logical combination (AND or XOR) of two of the four possible cues. We also varied the size of the reward associated with the logical combination. We discovered that this latter manipulation was a much better predictor of perceptron performance than was the logical structure of the interaction between cues. This indicates that when perceptrons learn to match probabilities, they do so by assuming that each signal of a reward is independent of any other; the best predictor of perceptron performance is a quantitative measure of the independence of these input signals, and not the logical structure of the problem being learned.

## Introduction

A perceptron [1, 2] is a simple artificial neural network whose input units send signals directly to an output unit layer through weighted connections. Each output unit then uses a nonlinear activation function to convert its incoming signal into a response ranging from 0 to 1. Modern perceptrons [3] typically use the logistic activation function

$$a_{j}=\frac{1}{1+e^{-({net}_{j}{+\theta }_{j})}}$$ (1)

where *a*_*j*_ is the activity of output unit *j*, *net*_*j*_ is the incoming signal, and *θ*_*j*_ is the bias of output unit *j*’s logistic activation function. Perceptrons originally served as systems for assigning input instances to discrete categories [1, 2]; more recent accounts continue to describe them from this perspective [4–6]. These accounts note that perceptrons can only solve linearly separable categorization problems [7]; more complex problems require networks that include hidden units.

However, perceptrons are still important to particular research domains, such as animal learning. When we interpret perceptron outputs as probabilities, perceptrons provide important new insights to the animal learning literature [8–11]. Furthermore, there exists a formal equivalence between models of perceptron learning and mathematical accounts of classical conditioning [12–14]. In these accounts, one translates the neural network learning rule into the animal learning rule by operationalizing the notion of reward as follows: a single-output unit network is ‘rewarded’ when its output unit is trained to turn on, and is ‘not rewarded’ when its output unit is trained to turn off. From this perspective, a single-output unit perceptron can be viewed as a simple instance of a reinforcement learning network [15].

The current paper extends a previous study [16] that explored the ability of modern perceptrons to match probabilities. It used four different input units to represent the presence or absence of four different cues, each of which signaled a different probability of reward. Perceptrons learned to match these reward probabilities, so that when the presentation of a particular cue caused the perceptron to generate output activity that equaled the reward probability associated with the cue. Furthermore, when reward likelihoods changed in the midst of training, perceptrons quickly learned to match the new probabilities.

One limitation of this previous study was that only one input unit was turned on at any given time. A more realistic task involves presenting more than one cue at the same time, with each cue independently signaling a different likelihood of reward. For a perceptron to match probabilities in this situation, it must learn to combine these different sources of information. The purpose of the current paper is to explore probability matching of perceptrons when they receive multiple sources of evidence.

This paper proceeds as follows: First, it reports on the ability of perceptrons to match probabilities when presented simultaneous cues. Second, it relates the structure of these trained networks to a well-understood statistical model, logistic regression. Third, it explores probability matching under conditions that challenge perceptron abilities. Fourth, it considers the implications of the structure of these probabilistic perceptrons, from the perspective of pattern classification and from the perspective of animal learning experiments.

## Simulation 1: Independent cues

### Method

#### Network architecture and training set

We trained perceptrons comprised of a single output unit and four input units. The input units represented the presence or absence of four different cues. The use of four cues emulated the structure of the perceptrons studied in our previous research [16]; by using additional input units, one could easily train a perceptron to process more than four cues. A binary representation indicated the presence or absence of cues. For example, the input pattern (1, 0, 0, 0) indicates the presence of Cue A and the absence of all other cues. We used each of the sixteen possible cue configurations (given this binary coding) in a training set.

Each cue signals a probability of network reinforcement. The presence of Cue A indicated a reward probability of 0.20, while Cue B, Cue X and Cue Y each signaled reward probabilities of 0.40, 0.60, and 0.80 respectively. These values are identical to those studied previously [16]. In this first simulation, the probabilities associated with the various cues were conditionally independent of one another. That is, the reward associated with the presence of one cue was determined independently of the reward associated with the presence of any another cue; of course, if different cues signaled a reward, the network was only rewarded once.

We constructed five different training sets. Each training set consisted of 1600 different input patterns; we replicated each of the 16 different possible cue configurations 100 times in a training set. Each input pattern was either rewarded (desired output activity = 1) or not (desired output activity = 0). We determined the reward for each pattern using the following stochastic procedure: We processed each cue separately. If a cue was present, we generated a random number between zero and one, and rewarded the pattern if the random number was less than or equal to the probability of reward associated with the cue. When this procedure produced a reward for more than one of the present cues, the pattern was only rewarded once. There was no reward if all four cues were absent. In using this procedure, each of our training sets is a random sample from the population whose cue probabilities were given earlier.

By constructing the training sets with this procedure, the problem represented by a training set is not linearly separable, because different instances of the same input pattern are associated with opposite rewards. Furthermore, by processing each cue independently conditional independence between the four cues is established. Because of this conditional independence, the addition rule for probabilities [17] can be used to check the probability structure of a training set, because this rule provides the expected likelihood of reward when more than one cue is present in an input pattern. For instance, for a pattern that includes both Cues A and B, and excludes both Cues X and Y, the probability of reward is:

P(R|AB)=P(R|A)+P(R|B)−P(R|A)∙P(R|B) (2)

In Eq 2, the various probabilities are those that define the reward structure for the population (e.g. P(R|A) = 0.20 and P(R|B) = 0.40). We used *χ*^*2*^ tests to compare the probability of reward for each type of pattern in a training set to the expected probability generated by the addition rule for multiple cues. None of the training sets differed significantly from the expected values.

#### Network training

**Fig 1:**
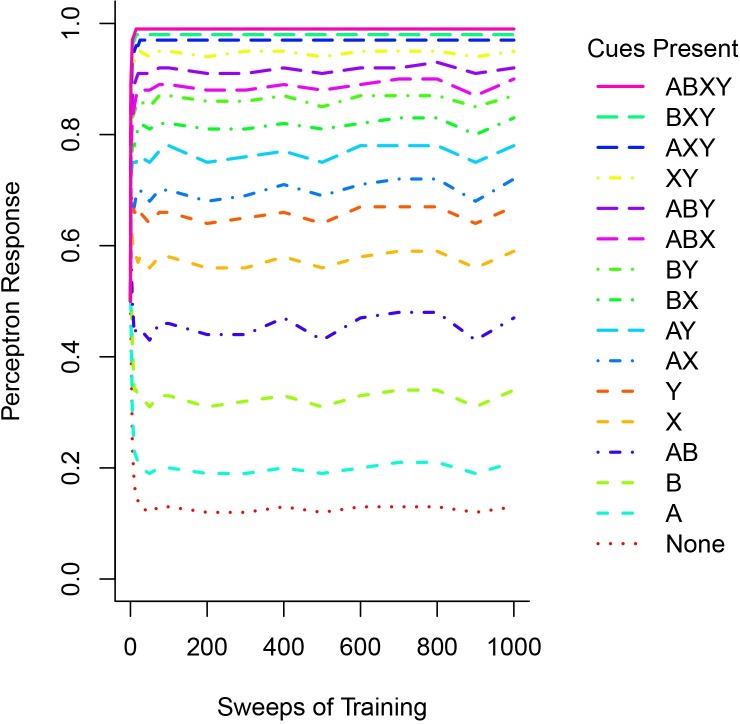
The responses of a perceptron to each of the 16 types of input patterns over the course of 1000 epochs of training. Responses are recorded after 1, 5, 10, 15, 20, 25, 50, 75, and 100 epochs, and then are recorded every 100 epochs until 1000 epochs of training have been conducted. The legend indicates which of the four cues are present in each of the 16 different stimulus patterns; the order of items in the legend matches the order of the lines as they are ‘stacked’ in the graph.

We trained 20 different perceptrons on each of the five training sets using a gradient descent rule with a learning rate of 0.05, with connection weights randomly set in the range from -0.1 to 0.1 prior to training, and with the bias θ of the logistic activation function initialized to 0. We chose a small learning rate of 0.05 to be able to examine the behavior of the network during learning (see Fig 1 below); even with a small learning rate, networks learned to match probabilities very quickly. We chose this learning rate to be small enough to make our learning algorithm a reasonable approximation of the mathematics of gradient descent, but at the same time to be large enough for network learning to succeed after a reasonable amount of training. However, this particular learning rate is not critical for our results; similar probability matching behaviors in networks occur when learning rates of 0.001, 0.1 or 0.25 are used.

Training was accomplished with the Rosenblatt program that is available as freeware [18]. During one epoch of training, we presented a network each of the 1600 patterns; the learning rule modified connection weights and the bias after each pattern presentation. We randomized the order of input pattern presentations every epoch. Training proceeded for 2500 epochs; we then recorded network responses to each of the 16 different possible input patterns, as well as the structure of the perceptron.

### Results

#### Network performance

Perceptron responses provide excellent estimates of the probability structure of the training sets. To assess probability matching, we computed the squared correlation (*R*^*2*^) between a perceptron’s responses to each of the 16 different types of input patterns and the actual reward probabilities for these patterns in the training set. Across the 100 perceptrons, the average *R*^*2*^ was 0.953 (*SD* = 0.014), indicating that typical network responses account for over 95% of the variance in the actual probabilities. Table 1 provides the performance of an example network (*R*^*2*^ *=* 0.97):

**Table 1:** Relation between the actual probability associated with each type of input pattern in one training set and a typical network’s responses to the patterns.

Conditional Probability	Input Pattern	Actual Probability	Network Response
P(R|~A~B~X~Y)	0,0,0,0	0.00	0.12
P(R|~A~B~XY)	0,0,0,1	0.63	0.65
P(R|~A~BX~Y)	0,0,1,0	0.64	0.57
P(R|~A~BXY)	0,0,1,1	0.90	0.95
P(R|~AB~X~Y)	0,1,0,0	0.35	0.32
P(R|~AB~XY)	0,1,0,1	0.88	0.86
P(R|~ABX~Y)	0,1,1,0	0.81	0.81
P(R|~ABXY)	0,1,1,1	0.98	0.98
P(R|A~B~X~Y)	1,0,0,0	0.21	0.19
P(R|A~B~XY)	1,0,0,1	0.79	0.76
P(R|A~BX~Y)	1,0,1,0	0.70	0.69
P(R|A~BXY)	1,0,1,1	0.96	0.97
P(R|AB~X~Y)	1,1,0,0	0.47	0.44
P(R|AB~XY)	1,1,0,1	0.94	0.91
P(R|ABX~Y)	1,1,1,0	0.77	0.88
P(R|ABXY)	1,1,1,1	0.96	0.99

The network performance provided in Table 1 indicates that its poorest response occurred when no cues were present. We found this overestimation in all 100 perceptrons. On average, for the no cue condition (actual probability = 0.00) network responses were 0.15 (*SD* = 0.02).

We explored this overestimation by creating ten new training sets. We used the method described earlier, with one exception: for five of the new training sets, the probability of reward when no cues were present was 0.05, while for the other five this probability was 0.10. We trained 20 new perceptrons on each of these new training sets. When the no cue reward probability was 0.05, network performance improved slightly over the results reported above (*R*^*2*^ *=* 0.962, *SD* = 0.009). When the no cue reward probability was 0.10, network performance improved slightly more (*R*^*2*^ *=* 0.973, *SD* = 0.011). However, more accurate estimates of the no-cue probability produced this improvement; there were no significant improvements observed for any other probability estimates.

Our previous research [16] demonstrated that perceptrons rapidly learned to match probabilities signaled by individual cues. To determine whether this was true with simultaneous cues, we examined perceptron responses to the 16 different input patterns at intervals of 5 epochs for the first 25 epochs of training, then at intervals of 25 epochs until 100 epochs of training had occurred, and then finally at intervals of 100 epochs until 1000 sweeps of training had occurred. We present th results for a typical perceptron in Fig 1. Prior to training, the perceptron generates activities of around 0.5 to each of the 16 patterns because of its small, randomly initialized weights. However, when training begins the responses of the perceptron quickly change to match the probabilities signaled by the various patterns. For instance, the perceptron illustrated in Fig 1 achieves probability matching (i.e. performance like that presented in Table 1) after between 25 and 50 epochs of training, and then maintains this performance for the remainder of training. There are slight variations in performance as training continues that result from presenting perceptrons patterns in random order.

#### Network interpretation

These results show that when different cues provide independent signals about reward probability, perceptrons can match probabilities with a high degree of accuracy. This suggests that the structure of a perceptron–its bias and its connection weights–implements the additive rule for independent probabilities. How does perceptron structure accomplish this? To answer this question let us consider the relationship between a modern perceptron and logistic regression [19].

Logistic regression is used to determine the relationship between a dependent variable and a set of independent variables when the dependent variable is dichotomous (typically coded as 0 or 1) [20]. It determines a set of coefficients (the *β*_*i*_ terms in Eq 3 below) that best predicts the probability that the dependent variable is equal to 1 given the states of the independent variables, which are also typically encoded as 0 or 1. Eq 3 provides an example of relating the probability of the state of the dependent variable (*Y*) to the states of four independent variables (*x*_*i*_):

$$P(Y=1|x_{1}x_{2}x_{3}x_{4})=\frac{e^{{(\beta }_{0}+\beta_{1}x_{1}+\beta_{2}x_{2}+\beta_{3}x_{3}+\beta_{4}x_{4})}}{1+e^{{(\beta }_{0}+\beta_{1}x_{1}+\beta_{2}x_{2}+\beta_{3}x_{3}+\beta_{4}x_{4})}}$$ (3)

Logistic regression uses a logit transformation involving the probability of the dependent variable [20]. This transformation takes the natural logarithm of the odds that the dependent variable has the state 1 given the states of the predictors; this transformation equals a weighted linear sum of the independent variables as is shown in Eq 4:

$$ln\;\left[\frac{P(Y=1|x_{1}x_{2}x_{3}x_{4})}{1-P(Y=1|x_{1}x_{2}x_{3}x_{4})}\right]=\beta_{0}+\beta_{1}x_{1}+\beta_{2}x_{2}+\beta_{3}x_{3}+\beta_{4}x_{4}$$ (4)

The conditional probability provided in Eq 3 is in fact a logistic equation [4–6]. This becomes obvious if we divide both the numerator and the denominator of Eq 3 by the numerator of the same equation, which produces the logistic equation in Eq 5. Note that this equation is identical to Eq 1 if we make the obvious assumption that *β*_*0*_ equals the bias θ, and that the sum of the remaining *β*_*i*_*x*_*i*_ terms is the net input, where each *β*_*i*_ is a weight and each *x*_*i*_ is an input activity.

$$P(Y=1|x_{1}x_{2}x_{3}x_{4})=\frac{1}{1+e^{{-(\beta }_{0}+\beta_{1}x_{1}+\beta_{2}x_{2}+\beta_{3}x_{3}+\beta_{4}x_{4})}}$$ (5)

Schumacher, Rossner and Vach [19] prove formal equivalence between a perceptron that uses the logistic activation function and logistic regression, as Eq 3 through 5 show. They also prove that, in principle, if we train such a perceptron using gradient descent, then its weights should be identical to the coefficients of a logistic regression of the same data. However, Schumacher et al note that, in practice, gradient descent training my not provide this result, particularly if it uses a constant learning rate. Furthermore, Schumacher et al.’s proof defines the error gradient over the sum of a network’s responses to all of the patterns in the training set (so-called batch training). Given that the Simulation 1 perceptrons use a fixed learning rate, and that they also learn via stochastic training which updates weights after every single pattern presentation (so-called stochastic training), whether these perceptrons match probabilities by converging on the same solution as logistic regression is an open question.

To answer this question, we performed a logistic regression for each of the training sets, using the *glm* function in R. Each logistic regression fit a model derived from the 2 X 2 X 2 X 2 contingency table for each training set. We then used *R*^*2*^ to assess the relationship between the five coefficients of the logistic regression and the five characteristics of network structure (i.e. the bias of the output unit and the connection weight associated with each cue). Each network’s structure was highly related to the regression coefficients; the average *R*^*2*^ across all networks was 0.99 (*SD* = 0.001). In short, each perceptron matches training set probabilities by adopting a structure that implements a logistic regression that maps cue signals into expected reward probabilities.

This finding is also important because it provides an elegant interpretation of each connection weight in the perceptron. If each of these weights is equivalent to a coefficient in a logistic regression (as demonstrated in the preceding paragraph), then each of these weights can be literally interpreted as the natural logarithm of the odds ratio associated with a cue. That is, the connection weight indicates how the odds of reward are altered by the presence or absence of a particular cue when the other cues are held constant [20]. We discuss the implications of this finding to the study of animal learning later in this paper.

## Simulation 2: Conditional dependence and linear nonseparability

In comparison to other pattern classification techniques, researchers typically dismiss perceptrons due to their inability to distinguish linearly nonseparable classes [4–6]. In Simulation 1, we considered perceptrons in the context of a different purpose, probability matching. Simulation 1 revealed that perceptrons are capable of performing this task even when facing linearly nonseparable problems.

However, perceptrons match probabilities in Simulation 1 because each cue is an independent signal of reward probability. If this independence is false, then perceptron performance will deteriorate. In logistic regression one would deal with conditional dependence by adding interaction terms to Eq 4 [20]. To do so with a perceptron requires adding additional input units to represent interactions between cues. How do departures from independence affect perceptron performance when we do *not* use additional units to encode interactions?

Simulation 2 explores this question by creating different training sets in which we hold constant the logical structure of a conditionally dependent relationship between Cues X and Y while we vary the amount of the interaction between them by manipulating the probability of reward that their interaction signals.

### Method

In Simulation 2, we create four different types of training sets using the same general method described for Simulation 1. All four types of training sets consist of 1600 patterns; in each training set Cues A and B are conditionally independent of all other cues, the presence of Cue A indicates a reward probability of 0.2, the presence of Cue B indicates a reward probability of 0.4, and the presence of no cues indicates a reward probability of 0.0. We replicate each of the 16 types of input patterns 100 times. The difference between the four types of training sets involves Cues X and Y.

In the first type of training set, Cues X and Y interact with one another because the reward that they signal depends on the states of both cues. In particular, the logical AND of X and Y signals a reward with a probability of 0.6. That is, *only* when both Cues X and Y are present do they signal this likelihood of reward. We created five different training sets of this type using a stochastic procedure analogous to that described in Simulation 1.

The second type of training set is identical to the first, with the exception that the AND of X and Y signals a smaller reward probability of only 0.1. Again, we generated five different versions of this training set. The purpose of this second type of training set is that it is logically identical to the first type (i.e. both have the same linearly separable relationship between Cues X and Y). However, by changing the likelihood of reward associated with this relationship, we vary the amount of interaction between the two cues (in probabilistic terms, we vary the amount of conditional dependence between the two cues).

In particular, consider one of these training sets. To operationalize the amount of conditional dependence, we first create two 2 X 2 contingency tables: one providing the frequency of occurrence of each combination of Cue X and Y when there is a reward, the other providing the frequencies of occurrence when there is no reward. Note that these two tables collapse across all instances of the independent Cues A and B. Second, we compute χ^2^ for both of these tables to measure the independence of Cues X and Y. Finally, we sum the two values of χ^2^ to operationalize overall ‘degree of conditional dependence’. As this sum increases, there is a higher amount of dependence between cues X and Y. For the five high reward probability training sets, the average of this sum was 75.08 (*SD* = 9.49). In contrast, the average sum for the five low reward probability training sets was 3.47 (*SD* = 2.04). This indicates that there is a much higher amount of conditional dependence between X and Y when their AND signals a reward probability of 0.6 than when their AND signals a reward probability of 0.1, even though type of relationship between X and Y is identical.

We also create two other types of training sets using a linearly nonseparable relationship between Cues X and Y. In the first, the XOR of cues X and Y signals a reward probability of 0.6. That is, these cues only signal this likelihood of reward when Cue X is present while Cue Y is absent, or vice versa. In the second, the logical XOR of Cues X and Y signals a lower reward probability of 0.1. Again, we generated five different versions of each of these two types of training sets.

Again, these two XOR-based training sets hold the relationship between Cues X and Y constant while varying the amount of conditional dependence between the cues. When the XOR of these two cues signals a reward likelihood of 0.6, the average sum of the χ^2^ values of the two 2X2 contingency tables involving X and Y was 273.09 (*SD* = 19.26), indicating a high amount of conditional dependence. In contrast, when the XOR of these two cues signals a reward likelihood of 0.1, the average sum of the χ^2^ values was 9.73 (*SD* = 4.60), indicating a lower amount of conditional dependence.

We trained 20 perceptrons on each of the five versions of the four different types of training sets described above, creating 400 different networks in total. We used the identical training procedure described in Simulation 1. At the end of training, we recorded perceptron responses to each of the 16 possible types of input patterns; we also recorded network structure.

### Results

As was the case for Simulation 1, we assessed the probability matching of perceptrons by computing the *R*^*2*^ between the actual reward probability associated with each input pattern in the training set and each network’s responses to these patterns. Table 2 provides the mean *R*^*2*^ for the 100 different perceptrons trained on each of the four types of training sets. For each of the four conditions summarized in Table 2 perceptron performance was poorer than that observed in Simulation 1. However, this was not because perceptrons failed to emulate logistic regressions for this data. In every case, perceptron structure was nearly identical to the corresponding logistic regression coefficients. The problem was that with only four input units the perceptrons could not capture the conditional dependence between Cues X and Y.

**Table 2:** The mean R^2^ (with standard deviations) between network responses and actual probabilities for 16 different input patterns in each of the four types of training sets.

	Reward Probability Equals 0.6	Reward Probability Equal 0.1
AND of Cue X and Cue Y	0.76 (0.03)	0.88 (0.03)
XOR of Cue X and Cue Y	0.28 (0.05)	0.87 (0.03)

An inspection of Table 2 reveals several interesting findings. First, on average networks perform better when there is an AND relationship between Cues X and Y than when there is an XOR relationship between these cues. Second, on average networks perform better when Cues X and Y combine to signal a lower probability of reward then when they combine to signal a higher probability of reward. Third, changing the probability of reward has a much larger effect on network performance for the XOR versions of the training sets than for AND versions of the training sets. An analysis of variance (ANOVA) of the data used to produce Table 2 confirms these general observations. There is a statistically significant main effect of the logical nature of a training set (XOR vs AND, *F = 4219*, *df = 1*,*396*, *p < 0*.*001*), a significant main effect of the degree of reward being signaled (high vs low, *F = 8796*, *df = 1*,*396*, *p < 0*.*001*), and a significant interaction between these two factors (*F = 3754*, *df = 1*,*396*, *p < 0*.*001*).

One problem with this ANOVA is that the logical structure of the relationship between Cues X and Y is confounded with conditional dependence. This is because when conditional dependence is operationalized using the sum of the χ^2^ metric, on average this metric is higher for the training sets based on XOR than it is for the training sets based on AND.

We can carry out two alternative analyses in light of this problem. First, we can simply predict the *R*^*2*^ fit between network responses and actual training set probabilities from the sum of the χ^2^ metric for each training set presented to each of the 400 perceptrons. This predicts network performance using degree of conditional dependence, and ignores the logical relationship between Cues X and Y. When we perform this analysis, we find that the degree of conditional dependence accounts for nearly all of the variance in the fit of network responses (*R*^*2*^ *= 0*.*983*, *F = 22990*, *df = 1*, *398*, *p < 0*.*0001)*.

We can contrast this result with a second analysis that predicts network performance using the dichotomous nature of the relationship between Cues X and Y (i.e. XOR vs AND). This result is significant as well, but only accounts for a quarter of the variance in network performance accounted for by our measure of conditional dependence (*R*^*2*^ *= 0*.*2458*, *F = 129*.*7*, *df = 1*, *398*, *p < 0*.*0001)*.

In short, the amount of conditional dependence provides a much better prediction of network performance than is provided by linear separability, though both are statistically significant predictors.

## General discussion

The purpose of the current study was to extend our previous research on probability matching in perceptrons. In the first simulation, we examined probability matching by signaling reward likelihood using four conditionally independent cues, any of which could be simultaneously present. All of the training sets in Simulation 1 were linearly nonseparable. However, by focusing on the predicted probabilities produced by perceptrons, rather than on separating rewarded patterns from those not rewarded, we discovered that perceptrons generated highly accurate responses. Perceptrons can match probabilities by processing multiple independent signals.

How do perceptrons accomplish such probability matching? We examined the bias and weights of networks after training, and compared them to the coefficients of logistic regression equations fitted to the same data. We found that the structure of the networks very nicely approximated the logistic regression coefficients. This relationship is expected in principle, but may not necessarily be achieved in practice [19]. Our results show that a variation of a gradient descent algorithm in which patterns are presented randomly, and in which network structure is updated after each pattern presentation [3, 18], is equivalent to performing logistic regression. Thus, our perceptrons learn to match probabilities by adopting weights that reflect the natural logarithm of the odds ratio associated with each cue, where each cue is an independent source of information about the probability of reward. The odds ratio associated with a cue in essence indicates the difference between the probability of reward when the cue is present and the probability of reward when the cue is absent. The net input to the output unit provides the sum of these ratios for all cues that are present, and the logistic activation function literally transforms this sum into the expected probability of reward.

The preceding paragraph provides a computational account of how perceptrons match probabilities. In cognitive science, other accounts, capturing different kinds of generalizations, are also required [21, 22]. In particular, implementational accounts of how probability matching occurs in the brain are required. Current research suggests that synapses in the brain are themselves highly stochastic, and pre- and post-synaptic mechanisms that modify synaptic efficacy via learning (e.g. long-term potentiation and short-term depression) can quickly modify a synapse’s stochastic behavior [23, 24]. This raises the possibility that such mechanisms of synaptic plasticity are well suited as implementational accounts of probability matching, particularly given our observation that probability matching is established quickly (Fig 1). Future research should explore the relationships between computational accounts such as the one developed in the current paper and implementational accounts like that developed by [23, 24].

Our computational account of probability matching in perceptrons also predicts when networks will be poorer probability matches. In particular, if reward probability is signaled by interactions between cues, then the cues that interact are not independent of one another. In this case, perceptron performance will deteriorate, because the perceptrons are ‘wired’ to assume cue independence. Our second simulation explored this issue by manipulating the conditional dependence between Cues X and Y. In this simulation, these two cues were conditionally dependent (i.e., they interacted) because their logical combination signaled reward probability. In half of the training sets, we used logical combination AND (which is linearly separable), while in the other half we used the linearly nonseparable XOR. We also varied the amount of conditional dependence between the two logically related cues. We accomplished this by having the interacting cues signal either a high probability of reward (0.6) or a low probability of reward (0.1). There is a lower amount of conditional dependence when there is a low probability of reward.

The results of Simulation 2 revealed, as expected, that the presence of conditional dependence between cues decreased perceptrons’ ability to match probabilities. However, this ability was still surprisingly good. For instance, while conditional dependence was present in all of the training sets used in this study, perceptrons were able to fit 75% or better of the actual variance in the training data in three of the four conditions (see Table 2). Furthermore, while linear separability (i.e. the logical relationship between Cues X and Y) was a significant predictor of perceptron performance, we discovered that degree of conditional dependence is a much stronger predictor.

These results have interesting implications for the general notion of perceptrons as pattern processors. Perceptrons lack hidden units which places well-known limitations on their ability to classify patterns [7]. Modern treatments continue to dismiss perceptrons as pattern classifiers because they are limited to distinguishing linearly separable classes [4–6]. However, perceptrons are capable of computing functions other than the digital classification of patterns. When faced with the analog function of judging likelihood of reward (or of class membership) they generate excellent performance even when classes are not linearly separable, as demonstrated in Simulation 1. This performance exploits conditional independence rather than linear separability. Simulation 2 revealed that when conditional independence was not true, perceptron performance could still be reasonably high, depending upon the amount of conditional dependence. Indeed, amount of conditional dependence was a much better predictor of performance than was linear separability.

In short, we agree that perceptrons have limits due to their structure. However, the nature of these limits depends upon the tasks they face. When trained to match probabilities, linear separability does not seem to be the appropriate metric to use to predict perceptron behavior. Other metrics, that measure the amount of conditional dependence, are more appropriate.

The current results also have very interesting implications for domains that can be informed by models based on perceptrons, such as animal learning [12]. One facet of this domain, contingency theory, relates strongly to probability matching.

Contingency theory attempts to explain how biological agents learn to estimate the causal contingency, or the probabilistic relationship, between cues and outcomes [25–36]. A typical contingency theory experiment is analogous to the tasks facing perceptrons in our two simulations: cues are paired (probabilistically) with outcomes, and subjects judge the strength of this relationship. Contingency theorists debate the kind of metric to use to measure or to define the strength of contingency [25, 26]. Of several possible measures one, called ΔP (which is a difference between conditional probabilities), arguably provides the best account of data. However, ΔP is problematic: for instance, it typically only applies to 2 X 2 contingency tables; we cannot compute it for a more complex design like the one used in Simulations 1 and 2.

Importantly, contingency theorists do not consider one alternative metric: the odds ratio [37, 38]. In general, an odds ratio is a ratio of odds, while odds themselves are ratios of probabilities. For example, they express the odds of reward when a particular cue is present relative to the odds of reward when that cue is absent.

Odds ratios are important in the context of this paper because logistic regression coefficients can literally be interpreted as being odds ratios [20]. As the structure of the perceptrons in our simulations emulates logistic regression coefficients, this means that we can also interpret perceptron weights and biases as odds ratios. Given that perceptrons have direct formal and empirical links to the animal learning literature [12], then this clearly indicates that contingency theorists should explore the potential of measuring contingency with odds ratios.
